# Chloropropanols and Their Esters in Food: An Updated Review

**DOI:** 10.3390/foods13182876

**Published:** 2024-09-11

**Authors:** Gizem Ozluk, Miguel Ángel González-Curbelo, Bulent Kabak

**Affiliations:** 1 Department of Food Engineering, Faculty of Engineering, Hitit University, Corum 19030, Turkey; 2 Departamento de Ciencias Básicas, Facultad de Ingeniería, Universidad EAN, Calle 79 no 11–45, Bogotá 110221, Colombia; magonzalez@universidadean.edu.co; 3 Biotechnology Laboratory, Machinery and Manufacturing Technology Application and Research Center, Hitit University, Corum 19030, Turkey

**Keywords:** chemical hazard, chloropropanol fatty acid esters, food safety, glycerol esters, glycidyl fatty acid esters, thermally treated food

## Abstract

Chloropropanols, their fatty acid esters, and glycidol and its fatty acid esters (GEs) are process contaminants in foods that pose potential health risks. These contaminants typically arise during the deodorization process of vegetable oils, particularly in high concentrations within oils like palm oil and products derived from them, such as margarine, baked goods, pastries, and infant formula. Chloropropanol esters and GE can hydrolyze under the influence of lipases, forming chloropropanols. Elevated temperatures during food production can lead to the release of free 3-chloro-1,2-propanediol (3-MCPD) or free 2-chloro-1,3-propanediol (2-MCPD) in products containing both fat and salt. The exposure to these contaminants, especially for infants and young children, raises concerns about potential health hazards. While extensive research has focused on 3-MCPD, 2-MCPD, and GE, knowledge regarding other chloropropanols such as 1,3-dichloro-2-propanol (1,3-DCP), 2,3-dichloro-1-propanol (2,3-DCP), and their fatty acid esters remains limited. This review aims to provide a comprehensive overview encompassing formation mechanisms, analysis methods, toxicological implications, occurrence patterns, exposure levels, mitigation strategies, and legislative considerations concerning these contaminants.

## 1. Introduction

Recently, there has been an increasing interest in the potential carcinogenic effects of contaminants that arise from heat treatment processes. Chloropropanols are a class of chemicals in which the hydroxyl group(s) of glycerol is replaced by chlorine. Based on the replacement position and the number of chlorines, it can be separated into 2-chloro-1,3-propanediol (2-MCPD), 3-chloro-1,2-propanediol (3-MCPD), 1,3-dichloro-2-propanol (1,3-DCP), and 2,3-dichloro-1-propanol (2,3-DCP) [1].

Since the inception of food processing, chloropropanol derivatives have been found in foods, but the free form of 3-MCPD was first identified in acid-hydrolyzed vegetable proteins (acid-HVPs) in 1978 [2]. The esterified derivative of 3-MCPD, conjugated with fatty acids, was identified in 1980 [3]. In 2006, the presence of 3-MCPD esters in refined vegetable oils was confirmed, and the fatty acid ester configurations of MCPDs were identified as process contaminants [4]. In December 2007, 3-MCPD esters were identified in high amounts in infant formulas [5]. Their presence was also observed in human breast milk [6]. Later, high quantities of 3-MCPD have been found in a wide range of heat-treated foods, including meat products, milk and dairy products, cereal-derived products, coffee, fried cheese, biscuits, and smoked foods [7]. While the formation of bound 2- and 3-MCPD, as well as glycidyl fatty acid esters (GEs), typically occurs during the deodorization process in the refining of oils and fats, free MCPD is usually produced during the process of roasting and smoking [8].

The presence of 1,3-DCP, 2,3 DCP, and 2-MCPD has been observed in a wide range of foodstuffs, although these compounds have received relatively less attention in research. Notably, 1,3-DCP has also been detected in drinking water [9], highlighting the importance of considering these substances. The comprehensive investigation of all three compounds was initiated in the early 2000s, with a primary focus on soy sauce [10,11,12].

Besides vegetable oils and foodstuffs, chloropropanols have also been recorded in several food contact paper products globally, causing significant concern. It has been reported that the primary origin of chloropropanols in food contact paper products is the residual substance from polyamide epichlorohydrin resin, which is a wet-strength agent frequently employed in pulp manufacturing. During this process, epichlorohydrin can undergo hydrolysis, resulting in the formation of 3-MCPD and 1,3-DCP [8]. Thus, 3-MCPD and/or 1,3-DCP may also be found in foodstuffs through migration from contact materials such as sausage casings, tea bags, and coffee filters [13].

GEs are considered food processing contaminants that are generated during the physical refining process of vegetable oils and fats at temperatures exceeding 200 °C, specifically during the deodorization step. Glycidol is produced from the hydrolysis of GE ingested through food consumption during human digestion [1].

Chloropropanols and their esters are of significant toxicological concern due to their potential carcinogenicity. Studies have shown that 3-MCPD, 2-MCPD, and DCPs (such as 1,3-DCP) can induce kidney and testicular toxicity in animal models [1], with 3-MCPD classified as a possible human carcinogen by the International Agency for Research on Cancer (IARC) [14]. Additionally, glycidol, a hydrolysis product of glycidyl esters, is recognized for its genotoxic and carcinogenic properties, posing a severe risk to human health [1]. The carcinogenic properties of glycidol were initially discovered through rodent studies conducted by the National Toxicology Program (NTP) in 1990 [15]. Chronic exposure to these compounds, especially through processed foods, highlights the importance of assessing and minimizing their presence in the food supply chain.

This review provides a comprehensive overview of carcinogenic chloropropanol derivatives, specifically MCPDs, DCPs, glycidol, and GE. This document provides an overview of the scientific research related to MCPD and GE formation mechanisms, occurrence, exposure, toxicological effects, analytical methods, mitigation strategies, and existing legislative frameworks.

## 2. Definitions, Properties, and Chemical Structures

**Figure 1:**
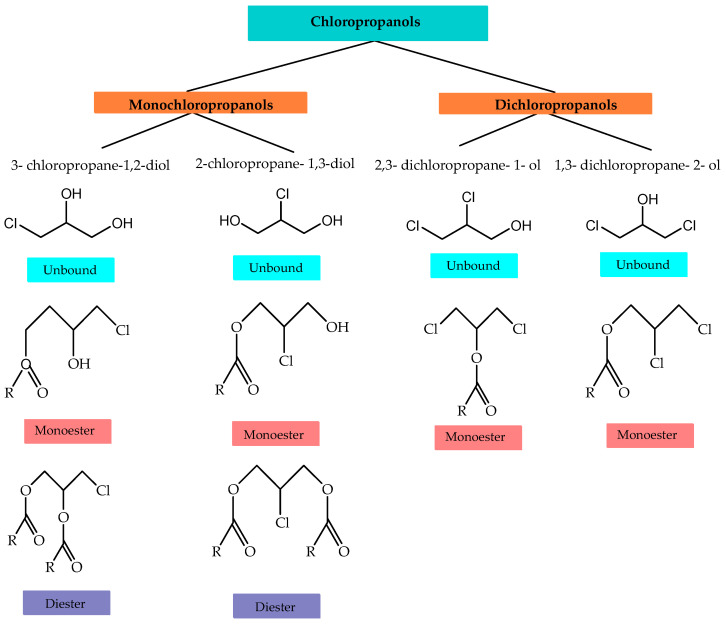
Chemical structures of chloropropanols and their esters.

Chloropropanols are chlorohydrins, which are propanols that include a chloride functional group. Chloropropanols are classified as monochloropropanol (MCPD) or dichloropropanols (DCP) based on the amount of chlorine atoms connected to the structure. Monochloropropanols are accessible in both monoesters and diesters, whereas dichloropropanols are only found in monoesters. They are designated after the location of the chlorine in the propanol molecule to which it is connected, such as 3-MCPD and 2-MCPD [1]. The chemical structures of chloropropanol isomers are shown in Figure 1.

These compounds are typically encountered in their esterified form with fatty acids. However, it is worth noting that they can also be found in small quantities in their free form. The chloropropanol esters are characterized by the presence of hydroxyl groups that have been esterified with fatty acids on the chloropropanol skeleton. MCPD-monoesters or MCPD-diesters are formed through the esterification of hydroxyl groups with fatty acids, either partially or fully. The compounds 3-MCPD, 2-MCPD, and glycidol are chloropropanols that are obtained from chlorinated derivatives of glycerol. The positioning of the chlorine atom differs between 3-MCPD and 2-MCPD. In 3-MCPD, the chlorine atom is located at position 3, whereas in 2-MCPD, it is situated at position 2 [9,16,17].

3-MCPD consists of three carbon (C) atoms, two functional alcohol (-OH) groups, and one chlorine (Cl) atom. The compound has a molecular formula of C_3_H_7_ClO_2_ and a molecular weight of 110.54 g/mol. The compound 3-MCPD is soluble in water, alcohol, diethyl ether, and acetone. It is colorless but may gradually turn straw yellow [18]. 3-MCPD has been classified as a rodenticide by the United States Environmental Protection Agency (US EPA) and is referred to as α-chlorohydrin [18]. 2-MCPD is an isomer of 3-MCPD; hence, the two have identical physical and chemical properties. 2-MCPD has a density of 1.3 g/cm^−^^3^, and it, along with 3-MCPD and glycidol, can be esterified with fatty acids [1,19].

Contrary to free MCPDs, the fatty acid esters of MCPDs are insoluble in water and polar solvents. The fatty acid esters of MCPDs can exist in nearly 100 different isomers due to the ability of the ester bonds to form at one or two of the available hydroxyl positions with any of the fatty acids. The number of GEs is extremely low due to the single hydroxyl group and the absence of positional isomers [1].

The compound 1,3-DCP consists of two chloride ions and has the chemical formula C_3_H_6_Cl_2_O. 2,3-DCP is an isomer of 1,3-DCP and includes two Cl ions. 1,3-DCP and 2,3-DCP are liquids at room temperature, with molecular weights of 128.98 g/mol and 163 g/mol, respectively. 1,3-DCP, which functions as a protic solvent and crosslinking reagent, is utilized as a solvent for hard resins and nitrocellulose. 2,3-DCP is utilized in the manufacturing process of epichlorohydrin. The utilization of 1,3-DCP and 2,3-DCP in chemical processes has raised concerns due to the potential for toxic effects resulting from the discharge of their waste into soil and water [20,21].

**Figure 2:**
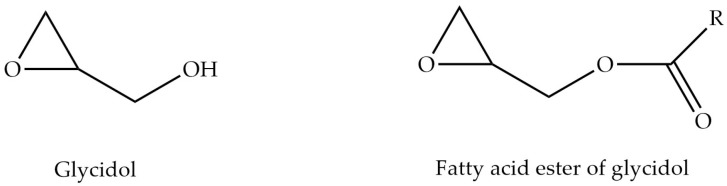
Glycidol and fatty acid ester of glycidol (R = alk(en)yl).

Glycidol, also known as 2,3-epoxy-1-propanol, is a colorless liquid that is soluble in water and most polar solvents. It has a molecular formula of C_3_H_6_O_2_ (Figure 2) and a molecular weight of 74.08 g/mol. It is an odorless liquid cyclic ether. Glycidol has a single hydroxyl group and forms only monoesters [1]. GE is another food process contaminant derived from refined oil. It contains an epoxide group esterified with fatty acids. Even though GE and MCPD esters are evaluated concurrently by researchers on account of their structural similarities, it is hypothesized that these compounds may exhibit distinct toxic effects because of the varying functional groups they possess [22,23,24].

## 3. Toxicological Effects

The toxicological impact of chloropropanols and their derivatives encompasses a range of mechanisms, including carcinogenicity, genotoxicity, and reproductive toxicity, observed across multiple species. The carcinogenic effects of chloropropanol and dichloropropanol were observed in rats, resulting in the development of various neoplasms. While chloropropanol’s genotoxicity was confirmed in vitro, dichloropropanol showed clear genotoxic effects in both in vivo and in vitro investigations. The specific method of action for the carcinogenicity of chloropropanol and dichloropropanol is unknown; however, evidence suggests probable hormone-mediated, cytotoxic, and genotoxic mechanisms [25]. The IARC has classified 1,3-DCP and 3-MCPD as “possibly carcinogenic to humans” (Group 2B). Additionally, glycidol has been classified as Group 2A, indicating that it is a probable carcinogen in humans [14,26].

It has been reported that the most important toxic effects of 3-MCPD are on the reproductive system [27,28,29]. 3-MCPD was previously considered a non-genotoxic carcinogen based on the observation that kidney tumors can arise from chronic nephropathy, and Leydig cell tumors can develop because of hormonal irregularities (known as endocrine disruptive effects) in the testicles [30,31]. It should be noted that 3-MCPD has been found to have toxic effects on the reproductive system and a potential carcinogenic effect on the kidneys, specifically renal tubular carcinoma [32]. It is effective in rats, rams, wild boars, guinea pigs, hamsters, rhesus monkeys, and human sperm but not in mice or rabbits [33]. Studies have demonstrated that 3-MCPD induces DNA strand breaks and causes genotoxicity in Chinese hamster ovary cells in vitro but does not cause DNA chain breaks in the liver, bone marrow, and lymphocyte cells in vivo [34].

The immunotoxic effects of 3-MCPD have also been recorded in female rats at a dose of 100 mg/kg body weight (b.w.)/day [35]. The various isomers of 3-MCPD exhibit distinct biological activities. The (S) isomer exhibits fertility inhibitory activity, while the (R) isomer has been found to have a detrimental effect on the kidneys [36].

A tolerable daily intake (TDI) of 0.8 mg/kg b.w./day for 3-MCPD and its fatty acid esters has been established, based on a BMDL_10_ value of 0.077 mg/kg b.w./day for the induction of renal tubular hyperplasia in rats, whereas no health-based guidance value could be established for 2-MCPD and its fatty acid esters [1].

The available information regarding the toxic effects of 2-MCPD is currently limited. The lethal dose (LD_50_) value for rats in the acute toxicity test was determined to be between 50–60 mg/kg [1]. It has been shown that 2-MCPD does not cause diuresis in Sprague-Dawley rats; however, 3-MCPD causes kidney failure and even renal failure [37]. No genotoxic potential has been observed for 2-MCPD [1]. The oral toxicological effects of chloropropanols and glycidol are summarized in Table 1.

Glycidol, like 3-MCPD, has been shown to cause infertility at low doses. This impact is assumed to be caused by the conversion of glycidol to 3-MCPD in the stomach. The rat’s lowest observed adverse effect level (LOAEL) for glycidol was determined to be 25 mg/kg b.w. per day. The administration of glycidol resulted in a reduction in both sperm count and motility in mice, with observations of testicular atrophy across all dosage levels. The LOAEL for mice was determined to be 75 mg/kg b.w. per day [1]. Terminal axon damage was observed when glycidol was administered to pregnant mice. It was found that the offspring of pregnant mice exposed to 1600 mg/kg glycidol had fewer interneurons. This shows that glycidole exposure during pregnancy may result in worse cognitive performance in kids later in life [42]. Upon exposure to a dosage of 200 mg/kg of glycidol, rats exhibited a gradual deterioration in gait abnormalities, as well as histological and immunohistochemical differences, and lesions appeared in the central and peripheral nerve systems. Glycidol has been observed to stimulate the growth of new neurons in the hippocampus and cause damage to axons during the later stages of development [43]. The compound has been reported to cause gene mutations and unscheduled DNA synthesis [44].

Glycidol has been shown to cause numerous genotoxic effects in mammalian cells, including genetic mutations, chromosomal abnormalities, sister chromatid exchange, and unprogrammed DNA synthesis. In addition to the reproductive system, investigations have revealed that glycidol can cause gastrointestinal, liver, and skin cancers when ingested orally. Considering the genotoxic and carcinogenic properties of glycidol, a margin of exposure (MOE) method was used. An MOE equal to or exceeding 25,000 was deemed to indicate a low level of health concern [1].

The LD_50_ values of glycidol for mice and rats were determined to be 431 mg/kg and 420 mg/kg, respectively [45,46]. Mihalache and Dall’Asta [47] calculated the potential cancer risk associated with glycidol, estimating an annual impact of 5.37/100,000 persons disability-adjusted life year (DALY). This suggests an elevated chance of developing cancer over one’s lifetime.

GE has been shown to have a lower genotoxicity potential than glycidol, and the MOE for individuals with average and highest exposure levels is specified as 17,800 and 10,900, respectively [48].

Based on the exposure data for 1,3-DCP and 2,3-DCP, The Committee on Muta-genicity of Chemicals in Food [49] concluded that the estimated 1,3-DCP intakes are of low concern to human health. Later, the Committee on Carcinogenicity of Chemicals in Food [50] reported that 1,3-DCP should be listed as a carcinogen. It has been observed that 1,3-DCP not only exhibits infertility effects but also induces the formation of tumors in the liver, kidney, ureter, and bladder. The oral LD_50_ values of 1,3-DCP and 2,3-DCP were 110 mg/kg and 90 mg/kg in rat, respectively [40]. These values are much lower than the specified value for glycidol, indicating that 1,3-DCP and 2,3-DCP have more acute toxicity than glycidol.

## 4. Formation

**Figure 3:**
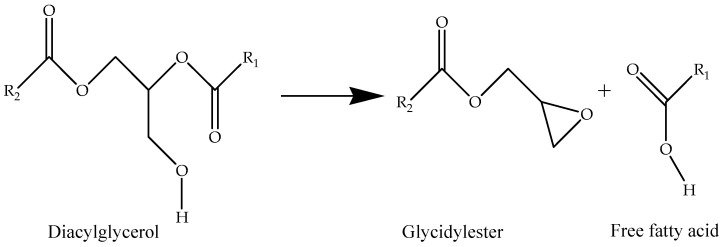
Possible formation mechanism of GE under thermal conditions.

Chloropropanols are known to be formed during food processing. The formation of 3-MCPD esters has been reported to occur through primary reactions during food processing in the presence of chlorine. This reaction occurs when foods that have a high fat and salt content are subjected to heat treatment. The synthesis of MCPD esters is primarily influenced by chlorine ions, acylglycerols, temperature, and time. On the other hand, GEs are formed under acidic conditions and high temperatures, primarily by monoglycerides and diglycerides [51]. The formation mechanism of GE under thermal conditions is illustrated in Figure 3 [52]. GE is mostly formed from diacetylglycerol during the deodorization process in the physical refining of oils, specifically at temperatures above 200 °C [1]. The hydrolysis of esters into their free forms, 3-MCPD and 2-MCPD, is facilitated by the lipase enzyme secreted by the pancreas in the intestinal system [53].

**Figure 4:**
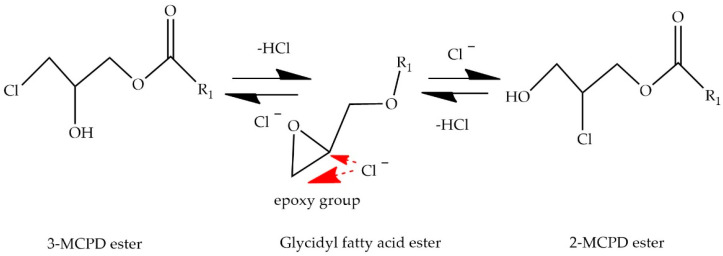
Conversion mechanism among 2-MCPD, 3-MCPD, and GE.

There are three hypothesized mechanisms for the formation of 3-MCPD in foods: acid hydrolysis, heat treatment, and lipase activity releasing it from its esters. 3-MCPD is generated through the chemical reaction between cooking oils and acid. The raw material contains HCl, triacylglycerols, phospholipids, and glycerol, which serve as precursors for the formation of chloropropanols [54]. The process of hydrohydrin production through the reaction of hydrochloric acid (HCl) and glycerol was initially documented by Velisek et al. [2]. In a later study, Hamlet et al. [36] put forward a mechanism that elucidates the heat-induced formation of chloropropanols from triacylglycerols in acidic environments. The crucial step in this mechanism entails the nucleophilic substitution of acyl groups with chlorine ions, which are activated by neighboring ether groups. The resulting intermediate is the chloropropanediol diester that yields chloropropanols under hydrolytic conditions. 3-MCPD can also be formed through a process of heat treatment in the absence of acid-HVP. The presence of this phenomenon has been observed in sodium chloride and lipid-containing foods during cooking methods such as grilling and baking. The release of free glycerol occurs through the high-temperature hydrolysis of triglycerides. This free glycerol has the potential to react with chlorine content [55,56]. Many researchers have demonstrated that 3-MCPD, 2-MCPD, and GE are formed during oil refining, and it has been stated that the deodorization process, particularly at high temperatures, plays an important role in their formation [56,57,58,59,60,61,62]. The conversion mechanism of 2-MCPD, 3-MCPD, and GE among each other is illustrated in Figure 4.

The generation of 3-MCPD increases with moisture content up to 30% but declines with increasing humidity. The production of 3-MCPD is influenced by pH in addition to temperature and humidity. It has been observed that 3-MCPD is not stable when the pH exceeds 6 [55].

Glycidol, an organic molecule with both epoxide and alcohol functional groups, can be synthesized through the process of dehalogenation from MCPDs [9]. Glycidol and GE are quickly absorbed following food consumption. After presystemic hydrolysis, GE quickly transforms to glycidol. Glycidol undergoes rapid metabolism through various enzymatic pathways, such as glutathione conjugation and mercapturate formation. The primary route of elimination for glycidol is urinary excretion [51].

3-MCPD esters are commonly produced alongside various other chloropropanol fatty acid esters, including 1,3-DCP and 2,3-DCP. 1,3-DCP, utilized as an intermediary in the manufacturing of epichlorohydrin, a raw material used in the chemical and paper industries, may be formed from hydrochloric acid and leftover lipids from the application material [9]. Another proposed mechanism for the formation of free 1,3-DCP is through the degradation of the substance by bacteria [63]. It has been proposed that 1,3-DCP can be formed through the direct conversion to glutathione conjugates or by metabolizing to β-chlorolactaldehyde, which undergoes oxidative dehalogenation and subsequent reduction to 3-MCPD [9,22].

## 5. Occurrence in Foods

Chloropropanols, their esters, and GE are commonly found in foods including hydrolyzed plant proteins, soy sausage, vegetable oils and fats, margarine, baked products, smoked foods, fried foods, infant formula, and other foods.

**Figure 5:**
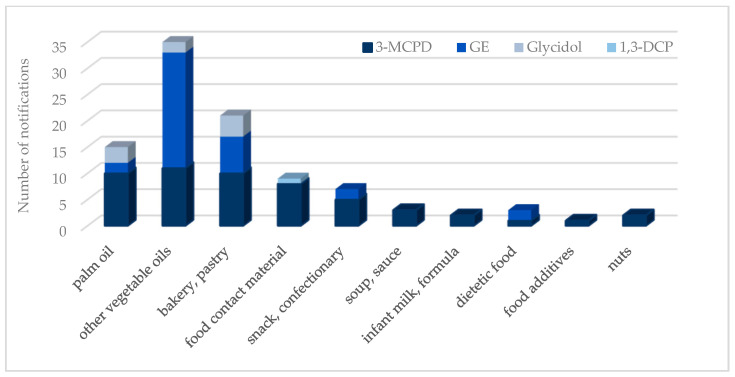
The numbers of RASFF notifications on chloropropanols, GE, and glycidol during the years 2020–2023 by product category.

The Rapid Alert System for Food and Feed (RASFF) reported a total of 54 notifications on 3-MCPD between the years 2020 and 2023. Most of these notifications were related to vegetable oils. A total of 36 notifications were documented for GE, whereas nine notifications were recorded for glycidol, and two notifications were recorded for 1,3-DCP. It should be noted that there were no recorded notifications for any chloropropanol compounds before the year 2020. The presence of chloropropanol has gained attention subsequent to the publication of the EFSA report. However, an analysis of the distribution of notifications over the years reveals a lack of comprehensive global efforts for mitigation. The number of RASFF notifications on chloropropanols, GE, and glycidol by product category during the period 2020–2023 is presented in Figure 5. Palm oil and other vegetable oils are the primary sources of food containing chloropropanol, followed by bakery and pastry products. The presence of 1,3-DCP is limited to food contact materials, while there is no available information regarding 2-MCPD and 2,3-DCP, potentially due to the absence of regulatory thresholds [64].

### 5.1. Vegetable Oils

The esters of chloropropanols, particularly 3- and 2-MCPD and GE, have been widely reported in various refined vegetable oils and fats, especially refined palm oil [65,66,67,68,69,70,71]. The occurrence and levels of fatty acid esters of 3-MCPD and 2-MCPD in vegetable oils from various countries are summarized in Table 2 and Table 3, respectively.

Among the vegetable oils, the highest middle bound (MB) values of 3-MCPD esters have been reported in palm oil/fat (2912 μg/kg), with >99 incidence, followed by palm kernel oil (100%, 624 μg/kg) and coconut oil (100%, 608 μg/kg). However, the MB levels of 3-MCPD esters in sunflower seed oil (93% incidence), maize oil (97%), soya bean oil (96%), rapeseed oil (84%), peanut oil (100%), and olive oil (100%) were 521 μg/kg, 503 μg/kg, 394 μg/kg, 232 μg/kg, 229 μg/kg, and 48 μg/kg, respectively. For 2-MCPD, the highest mean MB value was recorded in palm oil/fat (1565 μg/kg, 96% incidence) among the vegetable oil and fats group. Other vegetable oils and fats had a relatively lower MB level of 2-MCPD esters (86–270 μg/kg), with an incidence rate of 44–100%. For both fatty acid esters of 3-MCPD and 2-MCPD, the lowest MB values were recorded in olive oil [1].

Palm oil, classified as a fruit oil, has a higher water content compared to other seed oils, rendering it more susceptible to hydrolysis processes. These reactions lead to the formation of fatty acid esters of 3-MCPD and GE. The high 3-MCPD ester formation in palm oil was explained by the palm tree absorbing chlorine ions from the soil and water, which facilitates the formation of 3-MCPD. Furthermore, the synthesis of 3-MCPD esters in palm oil is directly proportional to its exposure to high temperatures during refining [77,78,79]. The study conducted by Razak et al. [73] demonstrated that the levels of 3-MCPD esters were higher in samples obtained from the bleaching and final stages of palm oil processing. In a recent study, Zhang et al. [80] discovered that the highest levels of 3-MCPD esters in palm oil can be attributed to the low activation energy required for the formation of 1,2-bis-palmitoyl-3-chloropropanediol.

The formation of 3-MCPD esters is influenced by several critical parameters, including the procedure of oil extraction from fresh fruit, fruit quality, chloride concentration, heating temperature, and heating time during the deodorization step of oil refining [72,73,76,78,80,81]. Moreover, a correlation between the presence of 3-MCPD and 1,3-DCP has been found in edible vegetable oils with a coefficient of 0.819 [82].

The occurrence and levels of GE in vegetable oils are summarized in Table 4. The highest GE level (18,000 µg/kg) was observed in the palm oil sample. Similarly, with a 99% incidence among the vegetable oil and fats group, palm oil/fat had the highest mean MB value for GE (3955 µg/kg) in the EFSA report. While no detectable level of GE was found in olive oil, the incidence of GE was 94% in maize oil (650 µg/kg), 92% in coconut oil/fat (mean MB = 476 µg/kg), 87% in palm kernel oil (421 µg/kg), 80% in sunflower seed oil (269 µg/kg), 70% in soya bean oil (171 µg/kg), 51% in rapeseed oil (166 µg/kg), and 50% in peanut oil (148 µg/kg) [1].

The two-fold higher concentrations of fatty acid esters of 3-MCPD, 2-MCPD, and GE values in the palm olein fractions were observed compared to the palm stearin fractions [67,73]. The data indicate that fatty acid esters of 3-MCPD, 2-MCPD, and GE tend to partition into the liquid phase, with a higher concentration found in the unsaturated fraction compared to the solid fraction. The explanation for this phenomenon is based on the physical properties of oils, which enable them to retain higher levels of fatty acid esters of 2-MCPD, 3-MCPD, and GE within their triacylglyceride structures compared to the solid fraction [67,73,83].

The concentration of MCPD esters increases with higher frying temperatures, while the GE content of oils decreases during the frying process. The impact of frying temperature on GE content is relatively insignificant while frying time has a more pronounced effect. The occurrence of glycidol from GEs at higher temperatures explains this phenomenon, resulting in a decrease in GE content and an increase in glycidol content during frying [84,85].

### 5.2. Foods Other than Vegetable Oils

Many studies have been conducted in the past two decades to investigate the concentrations of 3-MCPD, 2-MCPD, glycidol, and GE in various foodstuffs. The results of these studies are summarized in Table 5. The free and bound 3-MCPD values in other foodstuffs are significantly lower when compared to vegetable oils. However, the issue is in the excessive consumption of certain foods, which results in high exposure to compounds mostly because of the large quantities consumed rather than the actual levels of contaminants present.

In a survey by Chung et al. [13], a total of 318 different food samples consumed in Hong Kong, China, were monitored for the presence of chlorapropanols. The study revealed that approximately one-third of commonly consumed products were found to contain MCPD esters. Among various food categories, snack foods were found to have the highest levels of MCPD esters. The likely cause for this occurrence can be attributed to the oil content and the frying process employed in the production of snacks. 1,3-DCP esters were also found in 4% of the analyzed samples at lower concentrations (ranging from 1 to 9 µg/kg).

Ilko et al. [90] showed that the primary origin of 3-MCPD in potato products is the frying oils that are absorbed. Hence, it is reported that the concentrations of 3-MCPD, 2-MCPD, and GE are associated with the palm oils used as ingredients. In another study, Shi et al. [70] revealed a significant correlation between the levels of 3-MCPD esters or GE and the percentage of absorbed fat content in domestically oil-cooked food. Stauff et al. [88] measured the amounts of 2-MCPD, 3-MCPD, and GE in fine bakery products and found that these substances are present only in products made with refined vegetable oils. They also found that the GE levels, ranging from the limit of detection (LOD) to 1280 μg/kg, have been significantly decreased when compared to previous studies conducted on bakery products.

The presence of 3-MCPD has been detected in bread crust at levels of up to 400 µg/kg [97]. The formation of 3-MCPD increases when the toasted bread is exposed to high temperatures, resulting in an increased intake [55]. The formation of 3-MCPD compounds in commercial bread dough is primarily attributed to glycerol, which accounts for 68% of these compounds. During the baking process, chlorine ions and glycerol in yeast dough undergo a reaction with the precursor molecule, resulting in the formation of 3-MCPD [86].

The occurrence of chloropropanols in foods consumed by vulnerable groups, such as infants and young children, is of great concern. In the Czech Republic, 14 infant and baby food products were analyzed for the presence of 1,3-DCP, 3-MCPD, and their fatty acid esters. While none of the products contained free chloropropanols at levels above the method’s LOD, all of them contained high amounts of 3-MCPD esters. The concentrations of 3-MCPD esters in the infant and baby foods varied from 62 to 588 µg/kg, which corresponds to <300–2060 μg/kg on the fat basis [98].

In a United States survey from 2013–2016, 98 infant formula samples were tested for 3-MCPD esters and glycidol. The concentrations of 3-MCPD esters and glycidol in formulas containing palm and palm olein varied from 0.021 to 0.92 mg/kg and from less than the limit of quantification (LOQ) to 0.40 mg/kg, respectively. 3-MCPD esters and glycidol concentrations in formulas not containing palm and palm olein ranged from 72 to 160 μg/kg and from 5 to 150 μg/kg, respectively [91].

The presence of 3-MCPD in coffee is typically at very low levels, while coffee beverages do not contain detectable amounts of 3-MCPD due to the dilution effect of water [99]. The color of the coffee bean at the end of the process is closely linked to the formation of 3-MCPD and the presence of a high amount of dark beans. The presence of lipids and chlorine in coffee beans contributes to the formation of 3-MCPD during the roasting process [87,99]. Higher concentrations of 3-MCPD were detected in instant coffee, whereas the greatest concentrations were found in decaffeinated coffee and products that undergo extended roasting [89,100].

It has also been shown that smoked meat products contain 3-MCPD and its esters [101,102]. The level of 3-MCPD has been found to vary depending on the type of wood used for smoking and the duration of smoke treatment [103].

Air frying is an emerging frying technique that utilizes a newly designed appliance for home cooking. It involves the use of minimal or even no added oil for the frying process. A study was conducted by Ostermeyer et al. [104] to address concerns regarding the potential increase of chloropropanols resulting from the use of an air fryer. The effect of different domestic cooking procedures (baking in an electric oven, hot-air fryer, roasting in a pan, and roasting in a mini kitchen fryer) on the MCPD and GE amounts of fish fingers was determined. Sunflower oil was employed for the purpose of stir-frying, while frying oil was not utilized in the hot air fryer. The longer cooking duration in the hot air fryer resulted in a higher reduction in product weight compared to the oven. The levels of 3-MCPD and GE remain unchanged when food is cooked using a hot air fryer. The utilization of a hot air fryer can potentially impact the weight loss of food items under specific circumstances, although it appears to have minimal influence on their composition. The concentration of chloropropanol present in ready-to-eat frozen fish is influenced by the initial level of contaminants in the product and/or oil before cooking. On the contrary, Goh et al. [105] demonstrated that the use of an air fryer can still result in the production of 2- and 3-MCPD and GE, attributed to the elevated temperature and duration of the cooking process. In addition, the formation of chloropropanols can be facilitated by the presence of chloride ions, moisture, and partial acylglycerol.

Together with 3-MCPD, other chloropropanols such as 2-MCPD, 1,3-DCP, 2,3-DCP, glycidol, and GE have been found to occur in a range of foodstuffs. In addition to the presence of chloropropanol derivatives in foodstuffs, 1,3-DCP may also be found in drinking water. This is owing to its existence as a contaminant of epichlorohydrin-linked cationic polymer resins in flocculants used for water purification in a restricted number of treatment facilities [12]. The occurrence and levels of glycidol and chloropropanols other than 3-MCPD in foodstuffs are summarized in Table 6.

The available data regarding the content of 2-MCPD in food was primarily limited to soy sauces and oils before the publication of the EFSA report in 2016. It was observed that the level of 2-MCPD was lower than that of 3-MCPD, suggesting a potential correlation between these two contaminants. The levels of 2-MCPD were determined to be significantly low in dairy products, cereal products, meat, and fish. The presence of the substance was not detected in fruits and vegetables, confectionaries, bakery wares, salt, spices, soups and sauces (excluding soy sauce), coffee, and tea [12]. It has been reported that a correlation between 2-MCPD and 3-MCPD presence in various foodstuffs was found specifically in a 1:10 proportion [1]. Although free 2-MCPD is typically found in lower quantities than 3-MCPD in food, it exhibits greater resistance to the method used to reduce 3-MCPD [108].

It has been reported that all samples that tested positive for 1,3-DCP were also found to contain 3-MCPD. Additionally, the concentration of 1,3-DCP was noticeably lower compared to that of 3-MCPD. Meat, malt, and fish products were found to have very low quantities of 1,3-DCP, but cereals, spices, and sauces (excluding soy sauce) did not contain it [12]. However, Chung et al. [13] found higher amounts of 1,3-DCP in roasted pork and sausage than 3-MCPD. In another study, 1,3-DCP levels were found to be greater than 3-MCPD in cereals, snacks, toasted bread, and biscuits [109]. Zelinkova et al. [98] reported that the presence of 1,3-DCP was not detected in infant formula, follow-up formula, or growing-up milk. The presence of 1,3-DCP in food contact material, particularly in paper, has recently gained attention. This is due to the use of bleaching agents that contain chlorine [110].

## 6. Analytical Method

The analysis of chloropropanol derivatives is challenging due to their wide variety and structural complexity. There are two analytical approaches, namely direct and indirect, that can be used to determine 3-MCPD and related substances in foodstuffs [111,112].

The direct analytical approach is based on the quantitative determination of 3-MCPD and GE using liquid chromatography–mass spectrophotometry (LC-MS/MS) by analyzing each fatty acid that makes up the structure of mono and diglycerides separately. The analysis of GE involves purification using solid phase extraction (SPE) and gel permeation chromatography (GPC). The American Oil Chemists’ Society (AOCS) standardized the direct analytical method for determining GE in oils. However, the direct method for measuring 3-MCPD in food has not been fully validated. The fact that GE may be identified separately from MCPD and that the direct technique is simpler to use has increased the adoption of LC-MS/MS in recent years [113].

The indirect analytical approach is based on the quantitative examination by gas chromatography–mass spectroscopy (GC-MS/MS) of 3-MCPD and glycidyl components, which are esterified with fatty acid under acidic or alkaline conditions. Although several methods have been developed for indirect analysis, the purification, derivatization, and GC-MS/MS procedures are the same across all approaches. These approaches calculate the total MCPD or the sum of MCPD and GE in the food sample [108,114]. Although colorimetric and GC with flame ionization detection have been employed for detection in the air matrix, there is no currently appropriate method available for detecting unstable free glycidols in food [1].

The detection of 1,3-DCP and 2,3-DCP can be achieved using indirect methods, such as the elution of the compounds with a mixture of hexane and ether or ethyl acetate [55]. In another study, Cao et al. [115] developed a method for detecting 1,3-DCP and 2,3-DCP, as well as 3-MCPD, using 1-trimethylsilylimidazole as a silylating agent and GC-MS/MS.

In the analysis of chloropropanols and their esters, as well as glycidyl fatty acid esters, it is critical to report the LOD and LOQ to ensure the reliability and sensitivity of the analytical methods used. In chromatographic methods, the LOD for chlorapropanols and their derivatives can range from as low as 1 to 100 µg/kg [75,94,112,116,117,118,119], depending on the food matrix, sample preparation techniques, and specific analytical approach used (direct vs. indirect analysis). The LOQ for these contaminants typically falls within a slightly higher range, often reported between 5 and 200 µg/kg [1,75,76,94,112,117,119,120].

Apart from chromatographic techniques, a few approaches for the detection of chlorapropanols have been proposed, such as electrochemical and optical detections. Electrochemical methods, such as those using gold nanoparticle-enhanced sensors [121] and magnetic molecularly imprinted polymers [122], offer high sensitivity with detection limits down to 0.25 mg/L. Additionally, optical methods, including fluorescence-based assays on paper substrates [123], provide rapid analysis with LODs as low as 0.6 ng/mL. These methods complement traditional instrumental techniques by offering alternative approaches for detecting chloropropanols in food matrices.

## 7. Mitigation

Mitigating the formation of chloropropanols in food products is a complex but crucial endeavor involving a range of targeted interventions aimed at reducing their presence or preventing their formation during processing. Several strategies exist to prevent and/or reduce the formation of chloropropanols in foods. These strategies include the following:

- Removing precursor compounds from the raw material;
- Removing chloropropanols from the final product;
- Preventing chloropropanol synthesis by optimizing process parameters.

One precaution that can be performed before oil refining is the elimination of chlorine [115]. The chlorinated components present in crude oils can be effectively reduced by washing the oils with different solvents. The best results were obtained by washing the palm fruit pulp before oil extraction [52,124]. Additional precautions can be taken to mitigate the acidity that leads to the formation of contaminants. The acidity of the oil can be kept low by following certain precautions. These include carefully selecting the fruits that will be processed into oil, promptly collecting and sterilizing the fruits to deactivate the lipase enzyme, washing the fruits from palm trees that grow in acidic soil before processing them into raw oil, and storing the oil seeds at temperatures below 25 °C and with moisture content ideally below 7% [125,126].

Various adsorbents can be employed to eliminate chloropropanol derivatives from food. Zeolite and synthetic magnesium silicate resulted in a 40% reduction in MCPD esters [127]. An alternative approach involves the enzymatic conversion of 3-MCPD and its esters into glycerol using bacterial enzymes [128]. Additionally, the formation of contaminants can be prevented by introducing antioxidants to the oil. However, the inclusion of adsorbents, enzymes, and antioxidants appears to be impractical due to the need for supplementary procedural steps. However, the addition of antioxidants, such as α-tocopherol, stigmasterol, and squalene, to vegetable oil at higher concentrations led to the stimulation and increased formation of MCPD esters [129].

In recent years, researchers have concentrated on the anti-inflammatory, antioxidant, and chemopreventive characteristics of bioactive compounds, particularly those that protect against chloropropanol-induced toxic damage [130,131,132,133,134,135]. Flavonoids have been found to offer protection against the harmful effects of chloropropanols. Anthocyanins, notably cyanidin-3-glucoside, present in fruits and vegetables, have antioxidant characteristics and control gut microbiota, protecting against 3-MCPD-induced damage to the intestines and testis. Apigenin, found in fruits, vegetables, and plant-derived beverages, protects against 3-MCPD-induced kidney damage by altering mitochondrial activity. Furthermore, microRNAs, specifically miR-223-3p, play an important role in preventing acute kidney injury caused by 3-MCPD esters by inhibiting RIPK3 expression [131,132,133,134].

The CAC [126] advises implementing measures to reduce the levels of 3-MCPD esters and GE in refined oils and food products made with refined oils. These measures include following good agricultural practices (GAP), good manufacturing practices (GMP), and carefully selecting and using refined oils. It is recommended to reduce 3-MCPD formation in vegetable oil beginning with the early phases of vegetable plantation on the farm. Promoting the use of chlorine-free fertilizers, specifically supporting alternatives such as magnesium-rich synthetic gypsum (MRSG), may be beneficial for the low chlorine content of vegetables and oils [59]. Despite some positive developments in reducing contaminants, the desired outcome has not yet been achieved at the industrial level. Further investigation is required to reduce the chloropropanol formation in food products.

## 8. Legal Regulations

Several countries have established regulations for 3-MCPD and GE in hydrolyzed vegetable proteins, soy sauce, vegetable oils and fats, and infant formulas. The maximum levels (ML) of 3-MCPD in soy sauce in various countries are listed in Table 7. As can be seen in Table 7, the MLs of 3-MCPD in soy sauce vary from 20 µg/kg (in Europe, Turkey, and Malaysia) to 1000 µg/kg (in Japan, Canada, Taiwan, and the United States).

Worldwide, the most extensive regulations for 3-MCPD and GE have been established in the European Union (EU). For 3-MCPD, an ML of 20 µg/kg in hydrolyzed vegetable proteins and soy sauce has been established by Commission Regulation (EC) No 1881/2006 [145]. The original MLs for the sum of 3-MCPD and 3-MCPD fatty acid esters (expressed as 3-MCPD) and GE (expressed as glycidol) in certain foods, including specific food products for infants and young children were established by Regulation (EU) 2020/1322 [146]. The MLs in vegetable oils and fats, fish oils, and oils from other marine organisms are in the range of 1250 to 2500 µg/kg for 3-MCPD and 1000 µg/kg for glycidol. The MLs of 750 μg/kg 3-MCPD and 500 μg/kg glycidol are applied to vegetable oils and fats, fish oils, and oils from marine organisms that are specifically used to produce baby food and processed cereal-based food for infants and young children. The MLs of 3-MCPD and glycidol for baby formulae, follow-on formulae, food for specific medical purposes, and young-child formulae vary, with limitations of 125 and 50 μg/kg for powdered items and 15 and 6 μg/kg for liquids. However, the EU has reduced the MLs for the sum of 3-MCPD and 3-MCPD fatty acid esters in certain food products for infants and young children from 125 μg/kg to 80 μg/kg (for powder form) and from 15 μg/kg to 12 μg/kg (for liquid form). These new MLs will apply from 1 January 2025 [147].

No EU regulations were available for other chloropropanol derivates such as 2-MCPD, 1,3-DCP, and 2,3-DCP. The recommended MLs for 1,3-DCP in foodstuffs varied from 5 µg/kg in New Zealand and Australia [148] to 50 µg/kg in the United States and Switzerland [63].

## 9. Conclusions

Food products that contain carcinogenic chloropropanol derivatives can be consumed directly or used in the manufacture of other foods. The consumption of oils and foods containing these pollutants has been reported to be detrimental to health. Hence, it is crucial to implement measures to mitigate the occurrence of chloropropanol derivatives in food products and to eliminate any pre-existing contaminants. The presence of 3-MCPD in soy sauce has been well-known for decades, but a variety of foods have been shown to contain chloropropanol derivates. The EFSA report has highlighted increasing concerns regarding potential health risks associated with glycidol. The manufacturers focus their efforts on mitigating the presence of 3-MCPD and GE, as these substances have stringent restrictions. However, measures to reduce glycidol have not yet been put into action. The levels of exposure to 1,3-DCP and 2,3-DCP are quite low; however, the levels of GE have caused more significant concerns due to their larger concentrations. The utilization of 1,3-DCP and 2,3-DCP in chemical processes gives rise to concerns regarding potential toxic effects caused by the discharge of their waste into soil and water. It is important to give greater consideration not just to 3-MCPD but also to other chloropropanol derivatives.

In the future, research on chloropropanols and their esters is expected to focus on easier-to-use sample preparation techniques and detection methods, enhancing both sensitivity and specificity, especially for compounds like 1,3-DCP and 2,3-DCP. Efforts will also be geared towards identifying safer processing technologies to reduce the formation of these contaminants, possibly through novel enzyme treatments or alternative food processing methods that minimize high temperatures and the use of salts. Regulatory frameworks are expected to evolve in response to emerging toxicological data, resulting in stricter permissible limits and possibly new guidelines for previously overlooked contaminants, such as lesser-studied chloropropanols.

## Figures and Tables

**Table 1:** Oral toxic effects and the LD_50_ values of chloropropanols and glycidol.

Substance	Oral Toxic Effects	LD_50_, mg/kg b.w. (Species)	References
3-MCPD	Mutagenic effects on sperm Male infertility	191 (mouse) 152 (rat)	[38] [39]
2-MCPD	Toxic by ingestion	50–60 (rat)	[1]
1,3-DCP	Fetotoxic effects on reproductivity Tumorogenic effects on liver, kidney, ureter, and bladder	100 (mouse) 110 (rat)	[40] [40]
2,3-DCP	NA	90 (rat)	[40]
Glycidol	DNA damage in ovary and sperm Effects on embryo/fetus Male infertility Tumorogenic effects on gastrointestine, liver, skin, endocrine, and brain	431 (mouse) 420 (rat)	[41] [41]

**Table 2:** Occurrence and levels of 3-MCPD (from esters) in vegetable oils.

Product	Country	No. of Samples (*n*)	Incidence (%)	Range (µg/kg)	Method	References
Camellia oil	China	5	NA	988–2586	DGF method C-VI 18	[72]
Camellia oil (crude)	China	5	NA	250–555	DGF method C-VI 18	[72]
Canola oil	Malaysia	NA	NA	600	BfR Method 008	[73]
Maize germ oil	China	12	NA	219–1826	DGF method C–VI 18	[72]
Maize oil	Malaysia	NA	NA	250–300	BfR Method 008	[73]
Olive oil	China	11	100	34–1970	ISO-18363-4	[71]
Olive oil	Brazil	13	92	250–3900	AOCS Cd 29a-13	[74]
Olive oil (bland)	Brazil	17	35	250–620	AOCS Cd 29a-13	[74]
Olive oil (pomace)	Malaysia	NA	NA	1650	BfR Method 008	[73]
Olive oil (virgin)	Malaysia	NA	NA	350	BfR Method 008	[73]
Olive oil (virgin)	Brazil	46	17	250–1240	AOCS Cd 29a-13	[74]
Palm oil (bleached)	Malaysia	56	50	250–1800	BfR Method 008	[73]
Palm oil (crude)	Malaysia	105	20	250–900	BfR Method 008	[73]
Palm oil (refined)	Malaysia	NA	99	250–5800	BfR Method 008	[73]
Palm oil	China	18	100	270–8390	ISO-18363-4	[71]
Peanut oil (refined)	China	15	NA	450–1187	DGF method C-VI 18	[72]
Peanut/sesame oil	Malaysia	NA	NA	2450	BfR Method 008	[73]
Peanut oil	China	14	100	190–3720	ISO-18363-4	[71]
Rapeseed oil (crude)	China	9	NA	250–438	DGF method C-VI 18	[72]
Rapeseed oil (refined)	China	18	NA	226–1069	DGF method C-VI 18	[72]
Rapeseed oil	China	12	100	82–6400	ISO-18363-4	[71]
Refined vegetable oil	Singapore	36	NA	78–9592	AOCS Cd 29a-13	[70]
Rice bran oil	Malaysia	NA	NA	250–300	BfR Method 008	[73]
Sesame oil (crude)	China	6	NA	250–356	DGF method C-VI 18	[72]
Sesame oil (refined)	China	4	NA	651–1344	DGF method C-VI 18	[72]
Sesame oil	China	18	100	69–5860	ISO-18363-4	[71]
Soybean oil	Malaysia	NA	NA	<250	BfR Method 008	[73]
Soybean oil (crude)	China	7	NA	<250	DGF method C-VI 18	[72]
Soybean oil (refined)	China	18	NA	224–1090	DGF method C-VI 18	[72]
Sunflower oil	Malaysia	NA	NA	600	BfR Method 008	[73]
Sunflower oil (crude)	China	8	NA	<250	DGF method C-VI 18	[72]
Sunflower oil (refined)	China	6	NA	504–1044	DGF method C-VI 18	[72]
Sunflower oil	China	13	100	140–2580	ISO-18363-4	[71]
Vegetable oil (unrefined)	Singapore	24	NA	<30–1172	AOCS Cd 29a-13	[70]

NA: Not available.

**Table 3:** Occurrence and levels of 2-MCPD (from esters) in vegetable oils and fats.

Product	Country	No. of Samples (*n*)	Incidence (%)	Range (µg/kg)	Method	References
Edible oils	Poland	27	11	180–230	AOCS Cd 29b-13	[75]
Margarines	Poland	5	100	630–1700	AOCS Cd 29b-13	[75]
Palm oil	Germany	20	NA	200–5900	AOCS Cd 29b-13	[76]
Palm oil	China	18	100	150–6950	ISO-18363-4	[71]
Rapeseed oil	Germany	5	NA	<LOQ–300	AOCS Cd 29b-13	[76]
Rapeseed oil	China	12	100	71–4440	ISO-18363-4	[71]
Soybean oil	Germany	5	NA	<LOQ–300	AOCS Cd 29b-13	[76]
Soybean oil	China	21	100	336–1200	ISO-18363-4	[71]
Sunflower oil	Germany	5	NA	<LOQ–300	AOCS Cd 29b-13	[76]
Sunflower oil	China	13	100	28–1700	ISO-18363-4	[71]
Olive oil	Brazil	13	85	200–2010	AOCS Cd 29a-13	[74]
Olive oil (bland)	Brazil	17	12	200–280	AOCS Cd 29a-13	[74]
Olive oil (virgin)	Brazil	46	13	200–2160	AOCS Cd 29a-13	[74]
Olive oil	China	11	82	800–1010	ISO-18363-4	[71]

NA: Not available. LOQ: Limit of quantification.

**Table 4:** Occurrence and levels of GE in vegetable oils.

Product	Country	No. of Samples (*n*)	Incidence (%)	Range (µg/kg)	Method	References
Palm oil	Germany	20	NA	300–18,000	AOCS Cd 29b-13	[76]
Rapeseed oil	Germany	5	NA	<100–300	AOCS Cd 29b-13	[76]
Olive oil	Brazil	13	100	200–1910	AOCS Cd 29a-13	[74]
Olive oil (bland)	Brazil	17	100	200–1910	AOCS Cd 29a-13	[74]
Olive oil (virgin)	Brazil	46	35	200–2160	AOCS Cd 29a-13	[74]
Soybean oil	Germany	5	NA	<100–600	AOCS Cd 29b-13	[76]
Sunflower oil	Germany	5	NA	<100–400	AOCS Cd 29b-13	[76]
Vegetable oil (refined)	Singapore	36	NA	155–6204	AOCS Cd 29a-13	[70]
Vegetable oil (refined)	Singapore	24	NA	<30–1325	AOCS Cd 29a-13	[70]

NA: Not available.

**Table 5:** Occurrence and levels of free and bound 3-MCPD in various foodstuffs.

Substance	Product	Country	No. of Samples (*n*)	Incidence (%)	Range (µg/kg)	References
Free 3-MCPD	Cereal products	Czech Rep.	NA	NA	1–477	[86]
	Coffee	Czech Rep.	15	73	10–19	[87]
	Fine bakery	Germany	94	27	5–265	[88]
	Malt	Czech Rep.	22	32	10–95	[89]
Bound 3-MCPD	Cereal products	UK	NA	NA	3–2916	[86]
	Coffee	Czech Rep.	15	80	6–390	[87]
	Fine bakery	Germany	94	NA	18–1365	[88]
	Fried French fries	Czech Rep.	16	NA	100–258	[90]
	Infant formula	United States	98	NA	21–920	[91]
	Infant formula	Malaysia	16	100	2–244	[92]
	Potato crisps	Czech Rep.	56	NA	229–1008	[90]
Total 3-MCPD	Cereal and cereal products	Hong Kong	57	54	4–23	[13]
	Chicken seasoning cubes	Malaysia	6	100	90	[93]
	Dairy products	Hong Kong	12	0	<LOD	[13]
	Egg and products	Hong Kong	18	0	<LOD	[13]
	Fish and shellfish products	Hong Kong	66	22	3–33	[13]
	Fruits	Hong Kong	21	0	<LOD	[13]
	Infant formula	Brazil	40	37	<LOD–600	[94]
	Infant formula	United States	222	NA	13–950	[95]
	Meat and poultry products	Hong Kong	87	49	4–32	[13]
	Snacks	Hong Kong	24	38	6–66	[13]
	Soy-based sauces	Malaysia	43	54	<20–122	[93]
	Soy sauces	China	629	89	5–189,000	[96]
	Vegetable products	Hong Kong	39	0	<LOD	[13]

NA: Not available. LOD: Limit of detection.

**Table 6:** Other chloropropanol derivates than 3-MCPD in food.

Substance	Product	No. of Samples (*n*)	Incidence (%)	Range (µg/kg)	References
Bound 2-MCPD	Cereals	NA	NA	1–853	[86]
	Fine bakery products	94	NA	<3–624	[88]
	Infant formula	16	81	2–22	[86]
	Soy sauces	345	48	10–20300	[96]
1,3-DCP	Fish products	66	14	3–6	[13]
	Meat and poultry products	87	7	5–10	[13]
	Soy sauces	282	20	100–1400	[12]
	Soy sauces	345	19	4–8260	[96]
	Soy-based sauces	43	54	<LOD–25	[93]
	Water	>300		<100	[106]
	Water	24	NA	6–122	[107]
2,3-DCP	Soy sauce	71	10	13–28	[12]
	Soy sauces	345	4	3–500	[96]
GE	Infant formula		42	<LOD–750	[94]
	Infant formula	98	NA	5–400	[91]
	Infant formula	222	NA	19–370	[95]
	Infant formula	16	38	2–69	[92]
Glycidol	Fine bakery products	94	NA	<3–128	[88]

NA: Not available. LOD: Limit of detection.

**Table 7:** The ML of 3-MCPD in soy sauce in various countries.

Country	ML (µg/kg)	References
Australia	200	[136]
Canada	1000	[137]
China	400	[138]
European	20	[139]
Malaysia	20	[140]
New Zealand	200	[136]
South Korea	300	[141]
Turkey	20	[142]
United States	1000	[143]
Japan	1000	[48]
Taiwan	1000	[144]

## Data Availability

No new data were created or analyzed in this study. Data sharing is not applicable to this article.
